# Supplementary material for: Socioeconomic markers of dengue mortality in the 100 Million Brazilian Cohort (2007–2018): A nationwide registry-based cohort study
Source: PLoS Negl Trop Dis. 2025 Nov 3;19(11):e0013660. doi: 10.1371/journal.pntd.0013660 (PMC12582496; doi:10.1371/journal.pntd.0013660)
Supplement: S1 Table — Brazil. 2007–2018. (DOCX) [file pntd.0013660.s001.docx]

**Table S1.** Sensitivity analysis including only laboratory-confirmed dengue cases to assess socioeconomic factors associated with dengue-specific deaths and all-cause deaths within 15 days following the onset of symptoms of dengue. Brazil. 2007-2018.

| **Variables** | **Dengue-specific deaths** | | **All-cause deaths** | |
| --- | --- | --- | --- | --- |
|  | **P-value** | **OR crude (95% CI)** | **P-value** | **OR crude (95% CI)** |
| **Age (years)** |  |  |  |  |
| 20 - 39 |  | 1 |  | 1 |
| 0 - 4 | <0.001 | 2.33 (1.66-3.27) | <0.001 | 2.02 (1.46-2.80) |
| 5 - 9 | 0.181 | 1.20 (0.92-1.58) | 0.872 | 1.02 (0.79-1.33) |
| 10 - 19 | 0.132 | 0.86 (0.71-1.05) | 0.038 | 0.83 (0.69-0.99) |
| 40 - 59 | <0.001 | 1.42 (1.20-1.69) | <0.001 | 1.44 (1.24-1.68) |
| >= 60 | <0.001 | 2.60 (2.13-3.18) | <0.001 | 2.81 (2.35-3.36) |
| **Sex** |  |  |  |  |
| Female |  | 1 |  | 1 |
| Male | <0.001 | 1.45 (1.29-1.64) | <0.001 | 1.51 (1.36-1.69) |
| **Distal variables** | **P-value** | **OR adjusted* (95% CI)** | **P-value** | **OR adjusted* (95% CI)** |
| **Region of family home** |  |  |  |  |
| South |  | 1 |  | 1 |
| Northeast | <0.001 | 4.19 (3.11-5.64) | <0.001 | 3.61 (2.78-4.67) |
| Southeast | 0.319 | 1.16 (0.86-1.56) | 0.510 | 1.09 (0.84-1.41) |
| North | 0.008 | 1.65 (1.14-2.36) | <0.001 | 1.94 (1.42-2.63) |
| Midwest | <0.001 | 2.34 (1.73-3.19) | <0.001 | 2.06 (1.57-2.69) |
| **Location of the household** |  |  |  |  |
| Urban |  | 1 |  | 1 |
| Rural | 0.004 | 0.80 (0.68-0.93) | 0.012 | 0.83 (0.72-0.96) |
| **Race/ethnicity** |  |  |  |  |
| White |  | 1 |  | 1 |
| Black | <0.001 | 1.69 (1.36-2.09) | <0.001 | 1.61 (1.32-1.96) |
| Asian | 0.103 | 1.69 (0.90-3.16) | 0.081 | 1.67 (0.94-2.96) |
| Mixed Brown | 0.001 | 1.25 (1.10-1.41) | <0.001 | 1.25 (1.11-1.40) |
| Indigenous | 0.503 | 1.40 (0.52-3.77) | 0.459 | 1.40 (0.58-3.38) |
| **Proximal variables** | **P-value** | **OR adjusted** (95% CI)** | **P-value** | **OR adjusted*** (95% CI)** |
| **Level of education** |  |  |  |  |
| University graduate |  | 1 |  | 1 |
| Elementary and high school | 0.013 | 2.58 (1.22-5.44) | 0.016 | 2.16 (1.16-4.04) |
| Pre-school | 0.004 | 3.39 (1.48-7.75) | 0.003 | 2.92 (1.44-5.94) |
| Never went to school | 0.001 | 3.64 (1.70-7.78) | <0.001 | 3.13 (1.65-5.91) |
| **Employment** |  |  |  |  |
| Employed |  | 1 |  | 1 |
| Unemployed | 0.116 | 1.13 (0.97-1.32) | 0.152 | 1.11 (0.96-1.27) |
| Retired/pension | <0.001 | 2.03 (1.54-2.68) | <0.001 | 2.16 (1.69-2.74) |
| **Housing material** |  |  |  |  |
| Brick/masonry |  | 1 |  | 1 |
| Coated mud, wood, others | 0.998 | 1.00 (0.84-1.19) | 0.243 | 1.10 (0.94-1.28) |
| **Household water supply** |  |  |  |  |
| Public network connection |  | 1 |  | 1 |
| Water well, spring, others | 0.007 | 1.27 (1.07-1.52) | <0.001 | 1.37 (1.17-1.60) |
| **Sewage disposal system** |  |  |  |  |
| City public syst |  | 1 |  | 1 |
| Septic tank | 0.712 | 1.04 (0.86-1.25) | 0.732 | 1.03 (0.87-1.22) |
| Rudimentary cesspool, ditch, others | 0.037 | 1.18 (1.01-1.38) | 0.053 | 1.15 (1.00-1.33) |
| **Waste collection** |  |  |  |  |
| City collection |  | 1 |  | 1 |
| No collection, burned, buried, others | 0.234 | 0.87 (0.69-1.10) | 0.421 | 0.92 (0.74-1.13) |
| **Household density** |  |  |  |  |
| ≤ 2 inhabitants per room |  | 1 |  | 1 |
| > 2 inhabitants per room | 0.001 | 1.39 (1.16-1.68) | <0.001 | 1.37 (1.16-1.62) |
| OR: Odds Ratio |  |  |  |  |
| CI: Confidence Interval |  |  |  |  |
| The analysis was performed by excluding individuals with missing data from all models. *Covariates in distal model, for both models, were adjusted for age and sex. **For both outcomes (dengue-specific and all-cause deaths), covariates in proximal were adjusted for covariates from distal model with p<0.1 (i.e., region of family home, location of the household and race/ethnicity), age and sex. | | | | |
