# Supplementary material for: Socioeconomic markers of dengue mortality in the 100 Million Brazilian Cohort (2007–2018): A nationwide registry-based cohort study
Source: PLoS Negl Trop Dis. 2025 Nov 3;19(11):e0013660. doi: 10.1371/journal.pntd.0013660 (PMC12582496; doi:10.1371/journal.pntd.0013660)
Supplement: Attachment — Submitted filename: Reviewer_Report_PNTD-D-25-00509_Updated_june.docx [file pntd.0013660.s002.docx]

# Reviewer Report for PNTD-D-25-00509

Title: Socioeconomic risk markers of dengue mortality in the 100 Million Brazilian Cohort (2007-2018): A nationwide registry-based cohort study

Journal: PLOS Neglected Tropical Diseases

Manuscript Number: PNTD-D-25-00509

Reviewer: [Anonymous]

## Summary of the Manuscript

This manuscript evaluates the association between socioeconomic status and dengue mortality using data from the 100 Million Brazilian Cohort. The authors link socioeconomic, dengue, and mortality data from 2007–2018 to assess mortality outcomes within 15 days of dengue symptom onset. They apply a multivariable hierarchical analysis to identify both distal and proximal risk factors for dengue-specific and all-cause mortality. The findings indicate that individuals from socioeconomically disadvantaged backgrounds have significantly higher mortality risks.

## Major Strengths

- Large sample size (>3 million dengue cases) provides robust statistical power.

- High-quality data linkage using CIDACS-RL with verified accuracy.

- Methodologically rigorous with hierarchical modeling distinguishing distal and proximal variables.

- Valuable contribution to understanding socioeconomic disparities in dengue outcomes.

## Minor Comments

1. Improve consistency in terminology (e.g., 'pre-school' vs. 'illiterate').

2. Tables could be better formatted for readability.

3. Clarify how dengue diagnosis (confirmed vs. probable) influences the findings.

## Recommendation

I recommend: Minor Revision

The manuscript is of high quality and suitable for publication after minor clarifications and improvements.

1. Lack of control for comorbidities and pregnancy: The dataset used in the analysis does not include information on individual health conditions such as diabetes, hypertension, or pregnancy. These are well-established factors influencing dengue severity and mortality. Their exclusion could bias the estimation of socioeconomic effects.

2. Missing data on key variables: Variables like education (13% missing) and employment (10% missing) were excluded in complete-case analyses. This may reduce statistical power and introduce selection bias if the missingness is not completely at random. Sensitivity analyses with imputation or discussion of missingness mechanisms would strengthen the study.

3. Limited causal interpretation: Although the authors apply a hierarchical modeling framework, the observational design limits causal inference. There should be clearer discussion on how confounding and mediation are addressed and what assumptions are required for interpreting socioeconomic position as causal.

4. Uncertainty around diagnostic categories: The analysis includes both confirmed and probable dengue cases. It is not entirely clear how diagnostic certainty affects mortality estimates. Further elaboration on potential misclassification and its impact would be helpful.
