# Supplementary material for: Socioeconomic markers of dengue mortality in the 100 Million Brazilian Cohort (2007–2018): A nationwide registry-based cohort study
Source: PLoS Negl Trop Dis. 2025 Nov 3;19(11):e0013660. doi: 10.1371/journal.pntd.0013660 (PMC12582496; doi:10.1371/journal.pntd.0013660)
Supplement: Attachment — Submitted filename: Response to Reviewers.docx [file pntd.0013660.s003.docx]

**Socioeconomic markers of dengue mortality in the 100 Million Brazilian Cohort (2007-2018): A nationwide registry-based cohort study**

| **REVIEWER 1** | **ANSWERS** |
| --- | --- |
| Introduction. Please ensure all epidemiological data are properly referenced and verifiable, such as ‘In 2024, more than 14 million dengue cases and over 10,000 dengue-related deaths were reported globally.’ | Thank you for your observation. We have inserted the missing reference in the revised manuscript (page 5, line115). |
| Methods. Please describe in detail the exclusion criteria for this study population and whether non-endemic areas, short-term residence, and immunized persons were considered. | Thank you for your question. We did not exclude based on geographic region (endemic áreas), short-term residence, or vaccination status.  Regional differences were addressed in the analysis, by including geographic region as a covariate in the regression models.  We do not have data on short-term residence or dengue vaccination status but the vaccination was introduced only recently in the country. |
| Methods. The study outcome included all-cause deaths that occurred within 15 days of the onset of dengue symptoms, whether this included accidents. How the authors considered these causes of death. | Thank you for your comment. As described in the Methods section (page 9, lines 204-205), deaths with an underlying cause classified as external (ICD-10 codes V01–Y98) were not considered as fatalities. |
| Results. Since the authors viewed race/ethnicity as a distal factor in dengue mortality, affecting access to education, housing, and health care, which in turn is associated with dengue. This seems to be a framework for mediation analysis, and I'm curious about the results of the mediation analysis. | Thank you for your comment. We considered race to be a distal risk marker, and in our framework and we have not intended to investigate mediation. But rather understand the association between socioeconomic variables and dengue mortality. For this reason, we did not stratify our analyses by race.  Our goal was to explore the independent associations between socioeconomic factors and dengue mortality, using a hierarchical model to reflect the theoretical ordering of these variables. |
| Results. Sensitivity analysis, such as multiple interpolation, is recommended to improve the reliability of the results. | Thank you for your suggestion. We agree that multiple imputation is a valuable method for addressing missing data. However, in this study, the proportion of missingness in key variables such as education (13%) and employment (10%) was relatively low. To assess its potential impact, we conducted a sensitivity analysis using the Missing Indicator Method. In our case, all missing indicators were automatically omitted from the final model, most likely due to their low frequency and lack of association with the outcome. This suggests that the missing data had minimal influence on our estimates. We have added this explanation to the discussion (page 21, lines 412-414). Based on these findings, we decided not to apply multiple imputation. |
| Discussion. Elevated mortality risks for Black, Asian, and mixed-race individuals align with structural inequities, but the absence of adjustment for comorbidities (e.g., diabetes, hypertension) weakens causal interpretations. The authors acknowledge this limitation but should discuss its potential impact on OR estimates. | Thank you for your important comment. In this study, we were primarily interested in examining the total effect of socioeconomic variables on dengue mortality.  We agree that data on co-morbidities would have been valuable. However, we consider co-morbidities to be part of the explanation on how socioeconomic markers can be associated with dengue mortality. In the absence of this information, our results still provide insight into the overall association—including both the indirect effect and the direct effect not mediated by them. Therefore, while we acknowledge this limitation, we believe it does not weaken the interpretation (page 21, lines 417-420). |
| Discussion. It is recommended that the authors provide further discussion on the elevated case fatality rates within 15 days of dengue symptom onset for people who are retired or living on pensions. The authors mention that even after adjusting for age, the mortality rate is higher among those who are retired or living on pensions. This may have suggested that the effect of being retired or living on a pension is independent of aging. I think this is a novel phenomenon. | Thank you for your comment. We have expanded the Discussion section to explore this observation (page 20, lines 385-392). Even after adjusting for age, people who were retired or on pensions showed higher mortality, suggesting that retirement status may reflect other vulnerabilities, such as limited social support or barriers to timely healthcare. In Brazil, this group, especially among those receiving minimum-benefit pensions, often faces socioeconomic disadvantage, which may contribute to delays in recognizing disease severity and accessing appropriate care. |
| Discussion. The direction of the effect of place of residence when only laboratory-confirmed dengue cases are included contradicts the main analysis and requires further discussion. | Thank you for your observation. We have expanded the discussion to explore potential reasons for this change in the direction of the association (page 18, lines 345-352). In particular, we discuss how selection bias may explain the lower risk of mortality observed in rural areas when restricting the analysis to laboratory-confirmed cases. As laboratory testing is less accessible in rural regions and often reserved for severe or hospitalized cases, milder dengue infections may be underrepresented. This could result in a spurious protective association for rural residence in this subsample. |
| Discussion. Please propose specific measures for equitable access to health care and health improvement, and state who is responsible for them. Family doctors or the CDC? | Thank you for your valuable comment. We have revised the discussion section to include specific strategies to improve equitable access to healthcare and reduce dengue-related mortality, particularly among vulnerable populations (pages 22-23, lines 445-452). |
| Inconsistent formatting (e.g., “OR=1.58” vs. “OR = 1.58”) should be unified. | Thank you for pointing this out. We have carefully revised the entire manuscript and standardized the formatting of all statistical expressions. |
| **REVIEWER 2** | **ANSWERS** |
| The results are consistent and presented appropriately, I only suggest reviewing table 2.  Review the race/ethnicity variable number “1.021” in Table 2, as well as the employment variable number “1.106” | Thanks for the suggestion. We have now revised the table formatting. |
| We saw in the results that black and Asian individuals are more likely to die from dengue fever. In the discussion, this finding was attributed to structural racism and social conditions that affect black or mixed-brown people. And as for the Asian group, would it also be due to conditions similar to those of the other groups? | Thank you for your suggestion. We have incorporated information regarding the increased mortality among individuals self-identifying as Asian ethnicity into the Discussion section (page 19, lines 365–370). Although this population is small and diverse in Brazil, some subgroups may reside in rural or underserved areas and experience vulnerabilities such as limited access to healthcare, social invisibility, and regional disparities. These factors may partially explain the elevated mortality observed, though further investigation is warranted. |
| Why did you choose not to associate the black group with the mixed-brown group? | Thank you for your comment. We chose to present Black and Brown individuals as separate categories because they are distinct self-identified groups in the CadÚnico/IBGE classification, with potentially different socioeconomic profiles. This approach allows for a more nuanced analysis, although we recognize that both groups may share common structural vulnerabilities, which we address in the Discussion section. |
| **REVIEWER 3** | **ANSWERS** |
| Improve consistency in terminology (e.g., 'pre-school' vs. 'illiterate'). | Thank you for this helpful observation. We have now revised the terminology used to describe education levels. |
| Tables could be better formatted for readability. | Thank you for your suggestion. We have now revised the formatting of all tables to enhance readability and clarity. |
| Lack of control for comorbidities and pregnancy: The dataset used in the analysis does not include information on individual health conditions such as diabetes, hypertension, or pregnancy. These are well-established factors influencing dengue severity and mortality. Their exclusion could bias the estimation of socioeconomic effects. | Thank you for your important comment. In this study, we were primarily interested in examining the total effect of socioeconomic variables on dengue mortality.  We agree that data on co-morbidities would have been valuable. However, we consider co-morbidities to be part of the explanation on how socioeconomic markers can be associated with dengue mortality. In the absence of this information, our results still provide insight into the overall association—including both the indirect effect and the direct effect not mediated by them. Therefore, while we acknowledge this limitation, we believe it does not weaken the interpretation (page 21, lines 417-420). |
| Missing data on key variables: Variables like education (13% missing) and employment (10% missing) were excluded in complete-case analyses. This may reduce statistical power and introduce selection bias if the missingness is not completely at random. Sensitivity analyses with imputation or discussion of missingness mechanisms would strengthen the study. | Thank you for this important observation. We acknowledge that missing data in key variables, such as education (13%) and employment (10%), could potentially reduce statistical power and introduce bias if the missingness is not completely at random. To assess the potential impact, we tried to conduct a Missing Indicator Analysis including missing data as a category. However, due to the lack of deaths in those categories, this analysis was not possible to run. We have added this explanation to the discussion (page 21, lines 412-414). |
| Limited causal interpretation: Although the authors apply a hierarchical modeling framework, the observational design limits causal inference. There should be clearer discussion on how confounding and mediation are addressed and what assumptions are required for interpreting socioeconomic position as causal. | Thank you for this important observation. We acknowledge that, due to the observational nature of our study, causal interpretations must be made with caution. We did not perform a formal mediation analysis, as our goal was to estimate the association between socioeconomic variables and dengue mortality. To avoid implying causality, we revised the manuscript and replaced the term “risk” with “odds” where appropriate, to better reflect the associative nature of our findings. |
| Uncertainty around diagnostic categories: The analysis includes both confirmed and probable dengue cases. It is not entirely clear how diagnostic certainty affects mortality estimates. Further elaboration on potential misclassification and its impact would be helpful. | Thank you for this important comment. We agree that including both confirmed and probable dengue cases may introduce diagnostic uncertainty and potential misclassification. To assess the robustness of our findings, we conducted a sensitivity analysis restricted to laboratory-confirmed cases. The results were consistent in direction and magnitude for most variables, suggesting that the inclusion of probable cases did not substantially bias the estimates. The only notable exception was the variable for household location, where rural residence, which was associated with higher mortality in the main analysis, showed a protective association in the restricted model. We have added a discussion of this limitation and its potential impact to the revised manuscript (page 21, lines 421-427). |
