# Supplementary material for: Socioeconomic markers of dengue mortality in the 100 Million Brazilian Cohort (2007–2018): A nationwide registry-based cohort study
Source: PLoS Negl Trop Dis. 2025 Nov 3;19(11):e0013660. doi: 10.1371/journal.pntd.0013660 (PMC12582496; doi:10.1371/journal.pntd.0013660)
Supplement: Attachment — Submitted filename: Reviewer_Report_Accept.docx [file pntd.0013660.s004.docx]

# Reviewer Report

## Decision:

Accept

## Comments to the Authors:

I have carefully reviewed the revised manuscript entitled “Socioeconomic markers of dengue mortality in the 100 Million Brazilian Cohort (2007–2018): A nationwide registry-based cohort study.” The authors have addressed previous concerns and incorporated revisions appropriately. The study is methodologically rigorous, and the analyses are clearly presented. The conclusions are well-supported by the results and highlight the significant impact of socioeconomic inequalities on dengue-related mortality in Brazil.

The manuscript is now suitable for publication in PLOS Neglected Tropical Diseases. I recommend acceptance without further revisions.
