# Supplementary material for: Socioeconomic markers of dengue mortality in the 100 Million Brazilian Cohort (2007–2018): A nationwide registry-based cohort study
Source: PLoS Negl Trop Dis. 2025 Nov 3;19(11):e0013660. doi: 10.1371/journal.pntd.0013660 (PMC12582496; doi:10.1371/journal.pntd.0013660)
Supplement: Attachment — Submitted filename: Response_to_Reviewers_auresp_2.docx [file pntd.0013660.s005.docx]

**Socioeconomic markers of dengue mortality in the 100 Million Brazilian Cohort (2007-2018): A nationwide registry-based cohort study**

| **REVIEWER 4** | **ANSWERS** |
| --- | --- |
| Page 11/line 246: In the Results section, in the sentence “…using only the SIM records, dengue and dengue haemorrhagic fever were attributed as an underlying cause of death…”, the authors refer to the older classification of dengue cases. I recommend explicitly noting that, although dengue notifications now follow the revised clinical classification, deaths in the Mortality Information System (SIM) are still coded using the older ICD-10 categories (A90 and A91), which do not distinguish cases with warning signs from severe dengue. This clarification would help readers understand that analyses based on SIM data reflect the older classification system. | Thank you for your comment. We have added the ICD codes to clarify that analyses based on SIM data reflect the older classification system. |
| Page 15/line 289/290: Looking at the table 3, the variables living in rural areas as well as self-identifying as Asian does not seems to be statistically significant. | Thank you for this observation. We acknowledge that the association was not statistically significant for dengue-specific deaths. However, the result was statistically significant for all-cause mortality within 15 days of dengue symptom onset. We have highlighted this. |
| Page 19/line 365: The authors draw attention to a purportedly increased all-cause mortality among individuals self-identified as Asian. It would be important to reference additional studies that have reported similar findings. Although this result appears atypical, it is borderline in terms of statistical significance (with the confidence interval very close to one) and, when weighed against the relevance of the other findings, does not seem to warrant particular emphasis. In this regard, it may be more relevant to highlight the higher all-causes mortality among individuals identifying as Brown (Pardo) compared to those identifying as White, given that Brown/mixed-race individuals constitute the largest group within the Brazilian population. | Thank you for your comment. We have revised the discussion to highlight the increased mortality among individuals self-identifying as *Pardo* and Black, which represents a more robust and relevant finding in the context of racial and social inequalities in Brazil. |
| Page 21/Line 406: In this section, the authors state that it was not possible to determine the number of infections per individual, nor which episode was the most severe. This statement is not entirely clear. Would it not be possible to identify, at least within the study period, those cases with other reported episodes of dengue (notified as probable dengue cases) in the SINAN database? | Thank you for this comment. Although some individuals can have multiple notified dengue episodes during their lives, a limitation of the linkage process was that we only extracted the notification that best matched our cohort data (i.e., with the highest similarity score). Although we understand that we might have underestimated the true dengue mortality within the cohort, it is unlikely that the notification that was linked to our cohort among multiple ones is differential depending on individuals demographic or socioeconomic status. This information has been included as a limitation in the Discussion section of the manuscript. |
| The authors used CadÚnico data to classify cases according to social, economic, and household characteristics. This group is already highly vulnerable from a socioeconomic perspective. This suggests that, if the entire Brazilian population and its wide inequalities in living conditions were considered, differences in lethality would likely be much more pronounced. It would be appropriate for the authors to include a comment on this effect in their discussion. | Thank you for this important observation. We have now added a comment to the Discussion to acknowledge this limitation and emphasize how our findings may underestimate the true magnitude of socioeconomic disparities in dengue case fatality rate at the population level. |
